# A comparison of isometric, isotonic concentric and isotonic eccentric exercises in the physiotherapy management of subacromial pain syndrome/rotator cuff tendinopathy: study protocol for a pilot randomised controlled trial

**DOI:** 10.1186/s40814-017-0190-3

**Published:** 2017-11-14

**Authors:** Rita Kinsella, Sallie M. Cowan, Lyn Watson, Tania Pizzari

**Affiliations:** 1 La Trobe Sport and Exercise Medicine Research Centre, School of Allied Health, College of Science, Health and Engineering, La Trobe University, Melbourne, Australia; 2 Physiotherapy Department, St Vincent’s Hospital, 41 Victoria Parade, Fitzroy, Melbourne, Australia; 3 Clifton Hill Physiotherapy, 111 Queens Parade, Clifton Hill, Melbourne, Australia; 4 University of Melbourne, School of Physiotherapy, Melbourne, Australia; 5 LifeCare Prahran Sports Medicine Centre, Prahran, Melbourne, Australia

**Keywords:** Shoulder pain, Shoulder impingement syndromes, Rotator cuff, Rehabilitation

## Abstract

**Background:**

Subacromial pain syndrome (SPS) involving rotator cuff tendinopathy is a common cause of shoulder pain and disability. Evidence suggests that structured physiotherapy may be as effective as surgery in this condition with significant improvements demonstrated in trials involving scapular retraining, rotator cuff strengthening and flexibility exercises. Most published programs typically utilise isotonic concentric and/or eccentric strengthening modes. Recently, immediate analgesic effects and muscle strength gains following heavy-load isometric exercises in lower limb tendinopathy conditions have been observed. It is pertinent to ascertain whether such outcomes can be replicated in SPS/rotator cuff tendinopathy. The primary aim of this study is to establish the feasibility of undertaking a full-scale randomised controlled trial (RCT) that compares the effects of isometric, isotonic concentric and isotonic eccentric rotator cuff contractions when used as part of a semi-standardised exercise-based physiotherapy program in patients diagnosed with SPS. The secondary aim is to explore potential trends or treatment effects of the exercise intervention.

**Methods:**

Thirty-six participants diagnosed with SPS will be randomised to one of three intervention groups and undergo a one-on-one exercise-based physiotherapy intervention, involving scapular and rotator cuff muscle retraining and strengthening. Each group will utilise a different mode of rotator cuff strengthening—isometric, isotonic concentric or isotonic eccentric. Rotator cuff tendon responses to isometric loading are not yet established in the literature; hence, individualised, progressive loading will be used in this pilot study in accordance with symptoms. The intervention will involve two phases: during Phase 1 (weeks 1–6) participants undertake the active group-specific physiotherapy treatment; in Phase 2 (weeks 6–12), they undertake a progressive, but no longer group-specific exercise program. To determine feasibility, an evaluation of key study parameters including (a) ease of recruitment (rate and number as well as suitability of the assessment algorithm), (b) adherence to all phases of the exercise intervention including home program compliance and logbook completion, (c) participant non-completion (drop out number and rate) and (d) adverse events (nature and number) will be undertaken. Secondary outcomes will measure immediate effects: (i) within-treatment changes in pain perception (verbal rating scale (VRS) and shoulder muscle strength (hand-held dynamometer) as well as longer-term changes: (ii) shoulder-related symptoms and disability (Western Ontario Rotator Cuff Index (WORC) and Shoulder Pain and Disability Index (SPADI)), (iii) perception of pain (11-point numerical rating scale (NRS), (iv) shoulder muscle strength (hand-held dynamometer) and (v) perceived global rating of change score. The immediate within-treatment assessment of pain and muscle strength will be undertaken in treatments 2 and 3, and the longer term measures will be collected at the primary (conclusion of Phase 1 at 6 weeks) and secondary (conclusion of Phase 2 at 12 weeks) end-points of the study.

**Discussion:**

The findings of this pilot study will permit evaluation of this study design for a full-scale RCT.

**Trial registration:**

Australian New Zealand Clinical Trials Registry, ACTRN12616001676404

**Electronic supplementary material:**

The online version of this article (10.1186/s40814-017-0190-3) contains supplementary material, which is available to authorized users.

## Background

Shoulder disorders are a leading cause of pain and disability in our society with one in three people experiencing shoulder pain at some stage in their lives [1, 2]. Recurrence is common and symptoms are often persistent, with 40–50% of patients reporting ongoing morbidity after 6–12 months [3] and 14% after 2 years [4]. Subacromial pain syndrome (SPS) is the most common of all shoulder diagnoses reported to general practitioners [5] and has been shown to be the most prevalent upper extremity disorder seen in working populations [6].

Extrinsic factors have been proposed as causing compression and/or abrasion of the bursal side of the rotator cuff tendons, mechanically impinged between the acromion or coracoid and the humeral head [7]. This traditional model is increasingly challenged with intrinsic rotator cuff pathology considered a more likely source of symptoms [8, 9], especially as cadaver studies have shown that rotator cuff pathology occurs more frequently within the internal substance or on the joint side of the tendon [10].

Despite a rising incidence of acromioplasty surgery worldwide [11], there is ongoing debate regarding the best treatment methods for patients presenting with SPS with both surgery and conservative management producing equivocal results [12–16]. Surgical intervention in this condition is costly both at the health-system and individual level. Indeed, subacromial decompression surgery has been shown to be associated with more time off work, while physiotherapy has been shown to be more cost-effective and associated with fewer adverse events [17]. It is therefore increasingly advocated that a course of physiotherapy be undertaken before surgery is considered.

Several researchers have investigated the most effective physiotherapy approach for SPS with some evidence to suggest that a structured exercise program is most suitable [18–22]. Heterogeneity of exercise interventions along with poor reporting of exercise protocols has prevented definitive conclusions being drawn regarding the optimum exercises and exercise parameters for the treatment of this condition in both general [18, 21, 23, 24] and working [25] populations. Hanratty et al. [21], in their systematic review and meta-analysis of exercise rehabilitation in SPS, identified that those trials where significant improvements in pain and function were demonstrated involved multiple different types of exercises including scapular stability training and targeted through-range rotator cuff strengthening and flexibility exercises. The mode of strengthening has generally been either isotonic concentric and/or eccentric in published programs.

Although eccentric exercises have been much advocated for the treatment of tendinopathy, particularly in the lower limb [26], there is limited and/or conflicting evidence to show that superior clinical outcomes are achieved with eccentric loading programs compared to other types of loading in the management of Achilles and patella [27] or rotator cuff [22, 28] tendons. More recently, the effects of isometric exercises in lower limb tendinopathy have been investigated [29–31] with Rio et al. [30] demonstrating improvements in pain and strength following heavy-load isometric contractions in patella tendinopathy. There remains, however, a paucity of research into the effects of isometric exercises in patients presenting with SPS.

Given that exercise is generally accepted as beneficial in the management of patients with SPS [32] and given our increasing understanding of intrinsic rotator cuff pathology rather than extrinsic bony compression associated with this condition, it is pertinent to ascertain whether greater clinical gains can be achieved with rotator cuff rehabilitation that utilises a specific type of muscle contraction. This is of particular clinical importance if the analgesic effect demonstrated in other tendons of the body following isometric contractions [30] can be replicated in SPS, where patients are frequently severely impaired by the pain and loss of function they experience, whatever stage along the continuum of tendon pathology they may be [33–36]. Findings from a small pilot study [37] suggest that low-load isometric exercises for rotator cuff tendinopathy may positively influence pain and tendon thickness but little has been established in the literature regarding rotator cuff tendon responses to varying isometric loads. Hence, the dosage in this present study will be semi-tailored, as per clinical practice, according to pain, severity and irritability.

The primary aim of this study is to establish the feasibility of running a full-scale randomised controlled trial (RCT) that compares the effects of isometric, isotonic concentric and isotonic eccentric rotator cuff contractions when used as part of a structured semi-individualised exercise-based physiotherapy rehabilitation program in patients diagnosed with SPS. To achieve this aim, an evaluation of key parameters including (a) ease of recruitment (rate and number as well as suitability of the assessment algorithm), (b) adherence to all phases of the exercise intervention including home program compliance and logbook completion, (c) participant non-completion (drop out number and rate) and (d) adverse events (nature and number) will be undertaken and used to inform the implementation of a full-scale RCT.

The secondary aim is to offer insights into any potential trends in treatment effects observed between the groups, to explore whether faster gains in pain, strength and therefore function are achieved from either of the three exercise interventions. To achieve this aim and facilitate sample size  estimations for a full-scale RCT, data will be collected using the selected clinical outcome measures at specific study time-points, with within-treatment and pre- and post-intervention differences evaluated across the three groups.

## Methods

### Study design

**Fig. 1:**
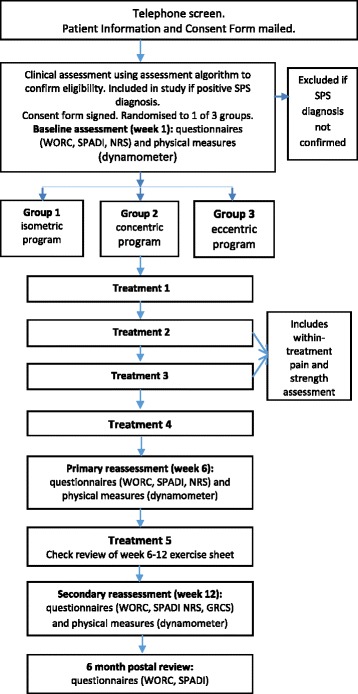
Study flow. *SPS* subacromial pain syndrome, *WORC* Western Ontario Rotator Cuff Index, *SPADI* Shoulder Pain and Disability Index, *NRS* numerical rating scale, *GRCS* global rating of change score

This protocol describes a pilot randomised, assessor- and participant-blind, controlled trial conforming to the SPIRIT 2013 [38] recommendations for clinical trial protocols. The study flow is outlined in Fig. 1. 

### Participants

Participants will be recruited from a physiotherapy outpatient clinic at a large public hospital and two private physiotherapy clinics, all within metropolitan Melbourne, using internal flyers and social media to promote participation. Since SPS is seen in both general and sporting populations, a combination of public and private sector recruitment sites will ensure a broader pool from which to draw participants. Based on a previous study [15] investigating the use of exercise in SPS with an effect size of 0.66 and maintaining a power of 0.80, calculations indicate a minimum of 30 patients would be required in each group for a full-scale RCT. To determine the feasibility for a full-scale RCT, a sample of 36 across the three groups (12 per group) has been chosen for this pilot study. This is approximately 30% of the calculation for a full-scale RCT [39] with an allowance for drop outs. It is anticipated that this sample size will provide the opportunity to observe recruitment rates using the assessment algorithm, adherence to and compliance with the various components of the intervention, number of participants lost to follow-up and number of adverse events; as well as enable preliminary evaluation of clinical outcome trends while saving the costs associated with a full-scale trial.

### Eligibility

**Fig. 2:**
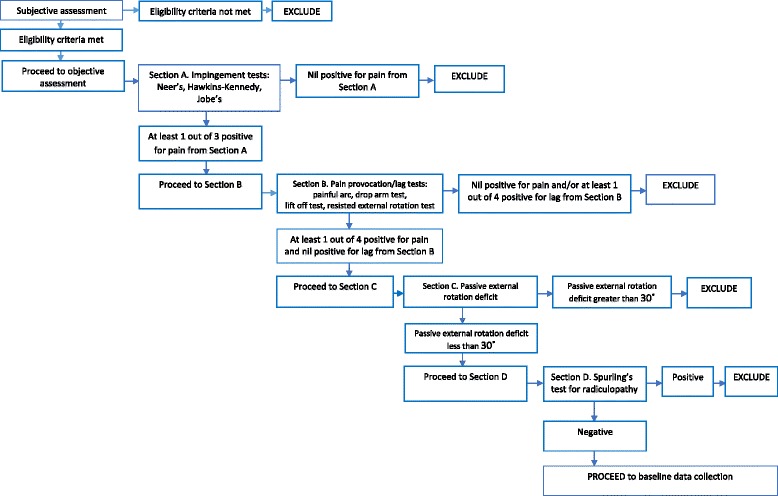
Assessment algorithm

There is no definitive diagnostic tool for SPS with no single impingement test shown to have high specificity or sensitivity [40, 41]. Further, a lack of consensus has been highlighted in the literature regarding participant eligibility criteria used in studies investigating this disorder [42]. Based on best available evidence [40, 41, 43–45], the combination of patient history and an assessment algorithm designed specifically for this pilot study will be used to assess eligibility. The assessment algorithm is outlined in Fig. 2 and is based on the following inclusion/exclusion criteria.

### Inclusion criteria

These include (i) aged 18–80 years (likelihood of patients > 80 having degenerative changes in the shoulder is increased), (ii) pain localised to the proximal anterolateral shoulder region, (iii) positive for pain on at least one of the following three impingement tests: Hawkins-Kennedy, Neer’s, Jobe’s and (iv) positive for pain on at least one of the following four tests: painful arc, drop arm test, lift-off test, and resisted external rotation.

### Exclusion criteria

These include (i) large, full-thickness rotator cuff tear, (ii) moderate-severe glenohumeral or acromioclavicular joint osteoarthritis, (iii) glenohumeral joint instability including previous shoulder dislocation/subluxation, (iv) previous shoulder fracture, (v) current neck pain/dysfunction with a somatic or radicular referral pattern indicative of cervical spine rather than shoulder as primary source of symptoms and/or pathology, (vi) neurological deficits of the upper limb and (vii) systemic inflammatory arthritic conditions.

Though patients routinely present with radiology including X-ray and ultrasound, which will be used to assist in diagnosis, inclusion/exclusion will be primarily based on clinical decision-making. Hence, where the patient meets the inclusion criteria outlined above, there must also be an absence of (i) positive rotator cuff lag signs (suggestive of a large rotator cuff tear), (ii) a passive external rotation range of motion deficit > 30° (suggestive of glenohumeral joint osteoarthritis) and (iii) a positive Spurling’s test (suggestive of cervical radicular/referred pain). To determine the severity of osteoarthritis, a shoulder X-ray undertaken within the previous 12 months is required for inclusion in the study.

### Procedures

Patients are referred to the public hospital physiotherapy outpatient clinic by their general practitioner or orthopaedic/other specialist. Those referred to the two private clinics may be referred by their general practitioner, orthopaedic specialist or via self-referral. Potentially eligible patients will be given information about the study. If they are interested in participating, their details will be passed to the study coordinator (RK) who will contact them to undertake a telephone screening interview. If still potentially eligible, the patient information and consent form will be mailed out and an appointment made for the patient to attend the clinic for further screening. Eligibility will be confirmed through a clinical assessment (using the assessment algorithm shown in Fig. 2) undertaken by a blinded assessing physiotherapist at each site.

Since there is a possibility of baseline differences in demographics between participants recruited from the public and private sectors, block randomisation will be used to ensure that participants from each of the clinics have an equal chance of receiving any of the interventions. Once consented, participants will be randomly allocated using an off-site randomiser and computer-generated allocation sequence to one of the three exercise groups: (i) isometric, (ii) isotonic concentric or (iii) isotonic eccentric. The treating clinician will be informed by the off-site randomiser via telephone of the group each participant is randomised to just prior to the commencement of the treatment intervention. While the treating physiotherapist cannot be blinded to treatment allocation, in order to minimise bias, the assessing physiotherapist will be blinded to group allocation, and patients will not be told which intervention group they have been randomised to.

The intervention will be carried out by designated, experienced physiotherapists at each site, with the treatment sessions delivered by a physiotherapist who is not involved in any stage of the assessment process. Prior to recruitment of participants into the study, all clinicians involved in assessment and treatment delivery will receive training in the assessment algorithm and treatment intervention along with all related procedures including treatment notes and documentation as per the study protocol. Treatment interventions including individual patient modifications will be recorded on standardised report forms. Participants will be provided with a logbook to record the number of home exercise sessions completed as well as adherence to the home exercise program. Adverse events and the use of co-interventions will also be recorded in the participant logbook. All adverse events will be documented by the treating physiotherapist and the project coordinator informed (RK). Monthly research staff meetings will be instigated for monitoring trial progress and to ensure prompt management of any issues that arise.

### Outcome measures

#### Primary outcome: feasibility of a full-scale RCT

The primary outcome of this study is to determine feasibility for a full-scale RCT. This will involve an evaluation of (a) ease of recruitment (rate and number as well as suitability of the assessment algorithm), (b) adherence to both phases of the exercise intervention including home program compliance and logbook completion, (c) participant non-completion (drop out number and rate) and (d) adverse events (nature and number). The regular monthly research staff meetings will provide an opportunity for continual evaluation to gauge whether the various components of the study work well together as well as allowing collection and monitoring of data relating to the key parameters that have been identified in (a–d) above.

In order to meet the target sample size, it is planned that the recruitment coordinator will achieve a telephone screening percentage of 75%, and the assessors at each site will achieve a clinical assessment screening percentage of 50%; screening will continue until the target population is reached (12 participants per site [38]. As making a diagnosis of SPS is complex [40, 41, 43–45], an assessment algorithm (see Fig. 2) has been designed in order to ensure the appropriate participants are included in this study [42]. Part of the feasibility of this study relates to the ease of use of the assessment algorithm by the assessors, their willingness to use it and its influence on recruitment rates. Calculating the time it takes to recruit will facilitate planning for the full-scale RCT.

Participant adherence will be monitored by recording the number of physiotherapy assessment and treatment sessions attended. For completeness of data collection and improved statistical analysis, we seek to maximise study retention and adherence. In accordance with the Pedro Scale criteria [46], we plan for a retention rate of at least 85%. By keeping the intervention period relatively short to reduce the patient burden as well as by contacting participants to remind them of their treatment and assessment appointments, we anticipate this will be achievable.

Compliance with the exercise intervention will be monitored via therapist logbook sign off at each treatment session. In studies that have investigated exercise interventions in participants with SPS, adherence to intervention protocols has been reported as 80% and over [47, 48]. We consider this will be achievable in our study with the exercise check-review during week 9, specifically designed to ensure ongoing compliance.

As well as participants recording adverse events in their logbook, further questioning regarding this will be undertaken by the assessor at trial completion. As all groups in this study will undertake an exercise-based intervention only, serious adverse events are not anticipated. Increased short-term pain during and following performance of exercises has been reported in other exercise-based studies [47]. As all of our participants will undergo a structured semi-individualised exercise program, with progression governed by symptoms and stage of tendon pathology, we anticipate minimal reporting of these kinds of minor adverse events.

#### Secondary outcome (i): immediate within-treatment changes in pain and strength

Since it is anticipated that the isometric exercise group may demonstrate greater immediate improvements in pain and strength, with a faster return to function, compared to either the isotonic concentric or isotonic eccentric groups, a key secondary outcome is to explore between-group within-treatment immediate changes in pain and strength during the rotator cuff strengthening component of the physiotherapy intervention. Measures of pain and strength will therefore be undertaken during treatment session 2 and 3 using a VRS (during shoulder motion) and hand-held dynamometer (resisted internal and external rotation) before and after completion of the intervention: a set of external (treatment 2) and internal (treatment 3) rotation contractions undertaken as per the group-specific contraction type (isometric/isotonic concentric/isotonic eccentric). All strength tests will be performed with the Commander Power track II hand-held dynamometer (JTech Medical). Each test will be performed as a “make” test, with the participant exerting a maximal isometric contraction against the dynamometer being held stationary by the tester [49–51]. For the dynamometer testing, participants will be tested in a standardised standing position—feet shoulder width apart, hips and knees in slight flexion, elbows flexed to 90° by the side of but not touching the body and wrist in neutral (palm facing midline) [49]. The test will be repeated twice with a rest of 5 s between tests [51].

#### Secondary outcome (ii): shoulder-related symptoms and disability

Shoulder-related symptoms and disability will be measured using the Western Ontario Rotator Cuff Index (WORC) and the Shoulder Pain and Disability Index (SPADI). Patient-reported outcome measures are increasingly recommended [52], and their use in patients with shoulder disorders have been investigated in several systematic reviews [53–56]. Both the SPADI and WORC have been shown to have good psychometric qualities that can be used for assessing individuals with shoulder pain including rotator cuff disorders [54]. Further, the WORC has been shown to be one of the most responsive questionnaires for patients suffering from rotator cuff disorders [54]. These outcome measures will be assessed at baseline, week 6, week 12 and via postal review at 6 months.

#### Secondary outcome (iii): perception of pain (current/usual/night)

Usual, current and night pain will be measured using an 11-point NRS. This will be undertaken at baseline, week 6 and week 12.

#### Secondary outcome (iv): shoulder muscle strength

Shoulder muscle strength will be assessed in varying test positions. As well as the within-treatment internal and external rotation in neutral test positions described above (secondary outcome (i)), three additional test positions will be undertaken at the main assessment points (baseline, week 6 and week 12). These will include shoulder abduction, external rotation at 90° and the empty can position, again using a hand-held dynamometer. Each test will be performed as a “make” test in the standardised standing position described above, held for 5 s and repeated twice [49–51].

#### Secondary outcome (v): perceived global rating of change score

Perceived change will be measured using a global rating of change score (GRCS) based on a 5-point Likert scale (much worse, slightly worse, no change, slightly better, much better) allowing patients to rate their perceived change following the intervention. Though criticised because of the need to recall baseline health status, GRCSs are commonly used to evaluate patient-perceived change in studies investigating interventions for shoulder pain [56] and have been shown to be clinically relevant, enabling interpretation of meaningful change in pain from a patient perspective [57]. The GRCS will be measured at the secondary end-point of the study (week 12).

### Physiotherapy intervention

The physiotherapy intervention will involve two treatment phases. In Phase 1, participants will attend on four consecutive weekly occasions for one-on-one treatment sessions with the physiotherapist. The intervention is exercise-based and aims to address (i) altered scapulo-humeral movement patterns, (ii) rotator cuff strengthening and (iii) upper quadrant flexibility/building posterior musculature (see Table 1). Participants will be taught the exercises at each visit with the intervention progressing in the following stages:

**Table 1:** Physiotherapy intervention overview

Treatment		Aim	Exercise	Description
Session 1	Retrain scapular movement patterns.	Scapular stability/retraining: optimise the position of the scapular and humeral head during shoulder motion, restoring normal scapulo-humeral rhythm.	Scapular setting. Scapular shrugs.	Scapular setting in standing at 0° with scapular rotation/ tilt correction (based on individual deficits). Build scapular stability-progression to scapular shrugs.
Session 2	Improve rotator cuff strength.	Improve rotator cuff strength (in optimal scapulo-humeral movement pattern).	Shoulder external rotation strengthening in neutral.	External rotation in standing with elastic (isotonic) or rigid (isometric) resistance band. Shoulder adducted to side and elbow at 90°.
Session 3	Improve rotator cuff strength.	Improve rotator cuff strength (in optimal scapulo-humeral movement pattern).	Shoulder internal rotation strengthening in neutral.	Internal rotation in standing with elastic (isotonic) or rigid (isometric) resistance band. Shoulder adducted to side and elbow at 90°.
Session 4	Build posterior musculature and restore flexibility.	Improve posterior muscle strength.	Standing rows at 45° or 90°.	Bilateral standing rows progressing from 45° to 90° shoulder abduction with resistance band.
		Improve flexibility of upper quadrant soft tissues.	Anterior shoulder stretch. Active thoracic extension. Lateral neck stretches.	Bilateral anterior chest stretch using room corner/door jamb. Sternal lift in sitting with lumbar-thoracic dissociation. Neck stretches in stand/sit.

Correction of the scapular or humeral head position (either by patient active self-correction or therapist manual correction) that improves symptoms during objective assessment tests forms the basis of treatment 1. Initial scapular “setting” exercises allow participants to develop the scapular stability required to ensure an optimal position of the glenoid, hence, a centred humeral head during shoulder motion. Scapular retraining will ideally be taught in a standing position, but since the cohort is likely to be heterogeneous, with varying scapular and humeral head static and dynamic motion deficits, alternative positions, aimed at minimising compensatory strategies, including prone and side-lie may be temporarily adopted. Though varying between individuals, in subjects with SPS, the scapular is frequently downwardly rotated and in anterior tilt [58]; thus, scapular upward rotation exercise drills including “modified shrugs” [59, 60] will be used in this phase to retrain the scapular stabilising muscles.

**Fig. 3:**
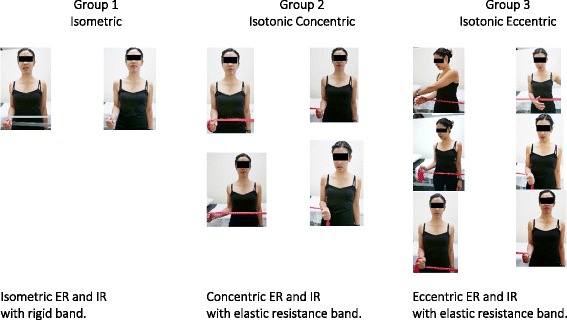
Rotator cuff strengthening exercises—between group variation, ER external rotation, IR internal rotation

The rotator cuff strengthening component of the physiotherapy intervention undertaken at treatment 2 and 3 will vary between the groups in terms of the specific type of muscle contraction taught—(i) isometric, (ii) isotonic concentric or (iii) isotonic eccentric (see Fig. 3). Taught in a standardised functional standing position, shoulder at 0°, the dosage will be semi-tailored according to pain severity and irritability, and in keeping with the tendon pathology continuum model [33]. Elastic resistance band will be used for the isotonic exercises (eccentric and concentric), while a rigid band will be used for the isometric exercises to ensure a static position is maintained.

Treatment 4 will address areas of relative flexibility including the thoracic spine and upper quadrant soft tissues as well as building posterior musculature. For the thoracic spine mobility exercises, retraining may be performed in varying positions, aimed at minimising compensatory strategies. Specific upper quadrant soft tissue flexibility exercises will include a bilateral anterior shoulder/chest stretch and lateral neck flexor stretches. Posterior muscle building will involve standing rows, taught and progressed at 45° and/or 90° determined by individual ability.

**Fig. 4:**
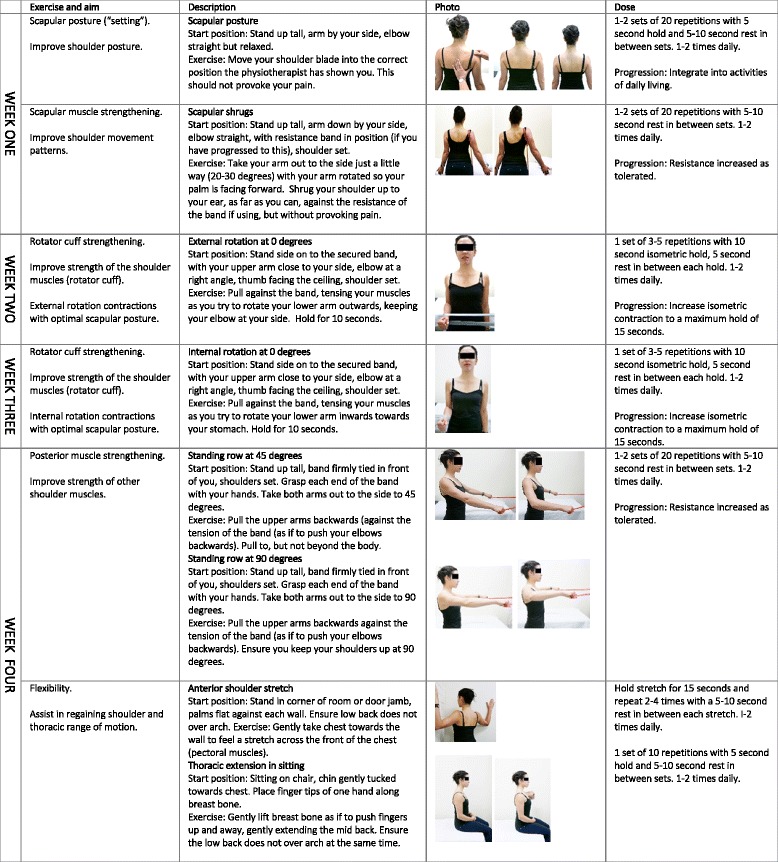
Phase 1 home exercise program—isometric group

Throughout Phase 1, home exercises based on the treatment intervention will be given with exercise progression as per individual response to load, aiming to maximise training effects. These will be performed independently and daily, dose dependent on exercise type. Participants will be provided with an exercise manual. Figure 4 outlines the Phase 1 home exercise program for the isometric group with the programs for the concentric and eccentric groups available as supplementary material [Additional files 1 and 2].

To standardise treatment as much as possible and since this study is primarily focussed on exercise intervention, manual therapy techniques will not be routinely used. Consistent with the usual clinical care of patients with this shoulder disorder, for individual participants who are unable to progress beyond an exercise stage without manual therapy to facilitate, this will be undertaken but kept to a minimum and recorded.

Phase 2 of the treatment intervention commences on completion of the 6-week follow-up assessment, when participants will be provided with an exercise sheet [Additional file 3] designed to progress them from 0° into range (and therefore no longer specific to one or more of the muscle contractions being evaluated in the trial). These exercises are based on the treatment intervention and home program. The patient will be instructed on how to perform the exercises that take them into a higher range of motion, focus on further strength gains and return to function. At week 9, they will attend a one-on-one check-review to ensure compliance with the exercise sheet.

### Data management and analysis

#### Primary outcome: feasibility of a full-scale RCT

Data collected on numbers of eligible participants recruited, numbers randomised via the assessment algorithm, adherence to and compliance with the intervention as well as drop outs lost to follow-up will be analysed as percentages and used to inform the development of a full-scale RCT.

#### Secondary outcomes: shoulder-related symptoms and disability (WORC, SPADI), strength (hand-held dynamometer), pain (NRS/VRS), perceived change (GRCS)

As this is a pilot study, it is not fully powered to determine treatment effects, and any inferential statistics will be used cautiously. Nevertheless, the analysis of between-group changes in secondary outcomes at each of the follow-up time-points may offer insights into possible trends and guide the design of a future full-scale RCT [61]. Similarly, baseline between-group participant characteristics and any associated influence on outcomes may also be observed. All data will be analysed as per-protocol. Analyses of variance (ANOVA) with repeated measures will be undertaken to evaluate trends in between-group changes in secondary outcome scores, with post-hoc analyses using Tukey’s HSD test performed where significant between-group differences are observed. Continuous variables (SPADI, WORC, 11-point NRS, VRS, hand-held dynamometer and GRCS) will be summarised using means and standard deviations, or medians and interquartile range, while categorical variables (gender) will be summarised using frequencies and proportions (and 95% confidence intervals). Analysis will be undertaken using SPSS statistical packages (Version 24, SPSS Inc. Chicago, IL) with estimation of effect sizes and confidence intervals; significance set at *p* < 0.05.

All data will be de-identified with analyses performed by an independent analyst. Groups will be coded and intervention allocation undisclosed so that the analyst is blind to the exercise program being used in any of the groups. Hard data will be stored in a locked cabinet and all soft files held on a password-protected computer accessible only to the research team. The principle investigator will have access to the final datasets. Results will be made available to participants on request and will be published in a peer-reviewed journal.

## Discussion

This manuscript describes a protocol for a pilot RCT that will compare the effects of isometric, isotonic concentric and isotonic eccentric rotator cuff contractions when used as part of a semi-standardised exercise-based physiotherapy program in patients diagnosed with SPS. Though research does suggest that exercise is an effective modality in the treatment of this sub-group of patients with shoulder pain [18–22], definitive evidence regarding which specific types of exercise, including intensity, duration and frequency, is lacking [21, 22, 25]. Similarly, though concentric and eccentric loading programs have been widely investigated and compared [22, 62–64], results remain inconclusive. Evidence is emerging on the benefits of isometric exercise in lower limb tendinopathy [30, 35], but there is limited research to date evaluating the effects of this mode of strengthening on rotator cuff tendons [37]. This study seeks to explore whether the isometric exercise group may demonstrate greater immediate improvements in pain and strength compared to either the isotonic concentric or isotonic eccentric groups, and as a result, potentially faster gains in function. Since a full-scale RCT is costly, this pilot study will evaluate whether the study design is feasible, in terms of ease of recruitment, suitability of the assessment algorithm, adherence and compliance to both phases of the treatment intervention, drop out rates and nature and number of adverse events. Increasing evidence suggests that a more individualised approach to therapeutic exercise is warranted in the management of SPS [65]. Since a cohort of patients presenting with SPS is likely to be heterogeneous, at varying stages of the tendinopathy continuum, this study may provide important preliminary information regarding treatment effect sizes of the semi-individualised physiotherapy intervention described, and enable more accurate power calculations for a full-scale RCT.

### Trial status

This trial is due to commence on 19th December 2016.

## Additional files

Additional file 1: Phase 1 home exercise program - concentric group. (DOCX 361 kb)

Additional file 2: Phase 1 home exericse program - eccentric group. (DOCX 385 kb)

Additional file 3: Phase 2 (week 6-12) exercise sheet. (DOCX 457 kb)

## Abbreviations

GRCS: Global rating of change scale
NRS: Numerical rating scale
RCT: Randomised controlled trial
SPADI: Shoulder Pain and Disability Index
SPS: Subacromial pain syndrome
VRS: Visual rating scale
WORC: Western Ontario Rotator Cuff Index
