# Supplementary material for: A comparison of isometric, isotonic concentric and isotonic eccentric exercises in the physiotherapy management of subacromial pain syndrome/rotator cuff tendinopathy: study protocol for a pilot randomised controlled trial
Source: Pilot Feasibility Stud. 2017 Nov 14;3:45. doi: 10.1186/s40814-017-0190-3 (PMC5684744; doi:10.1186/s40814-017-0190-3)
Supplement: Supplementary file 2 — Phase 1 home exericse program - eccentric group. (DOCX 385 kb) [file 40814_2017_190_MOESM2_ESM.docx]

Phase 1 home exercise program - eccentric group

|  | Exercise and aim | Description | Photo | Dosage |
| --- | --- | --- | --- | --- |
| **WEEK ONE** | Scapular “setting”.  Improve shoulder posture. | **Scapular posture**  Start position: Stand up tall, with your arm by your side, elbow straight but relaxed.  Exercise: Move your shoulder blade into the correct position the physiotherapist has shown you. This should not provoke your pain. | 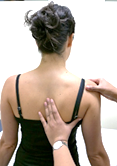 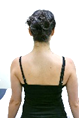 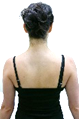 | 1-2 sets of 20 repetitions with 5 second hold and 5-10 second rest in between sets. 1-2 times daily.  Progression: Integrate into activities of daily living. |
|  | Scapular muscle strengthening.  Improve shoulder movement patterns. | **Scapular shrugs**  Start position: Stand up tall, arm down by your side, elbow straight, with resistance band in position (if you have progressed to this), shoulder set.  Exercise: Take your arm out to the side just a little way (20-30 degrees) with your arm rotated so your palm is facing forward. Shrug your shoulder up to your ear, as far as you can, against the resistance of the band if using, but without provoking pain. | 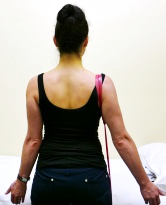 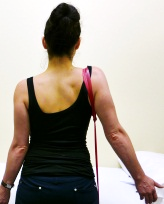 | 1-2 sets of 20 repetitions with 5-10 second rest in between sets. 1-2 times daily.  Progression: Resistance increased as tolerated. |
| **WEEK TWO** | Rotator cuff strengthening.  Improve strength of the shoulder muscles (rotator cuff).  External rotation contractions with optimal scapular posture. | **External rotation at 0 degrees**  Start position: Stand side on to the secured band, with your upper arm close to your side, elbow at a right angle, thumb facing the ceiling, shoulder set. Rotate your am outwards so that it is at 45 degrees.  Exercise: Take the exercise band in your non-treatment side and pass it across to your treatment side, enduring it is on tension.  Exercise: Allow your arm to return to the midline, controlling the band as you do so. | 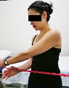 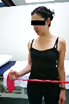 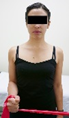 | 1-2 sets of 20 repetitions with 5-10 second rest in between sets. 1-2 times daily.  Progression: Start with arm rotated further outwards so that you have to control the band through greater range.  Progression: Resistance band increased as tolerated. |
| **WEEK THREE** | Rotator cuff strengthening.  Improve strength of the shoulder muscles (rotator cuff).  Internal rotation contractions with optimal scapular posture. | **Internal rotation at 0 degrees**  Start position: Stand side on to the secured band, with your upper arm close to your side, elbow at a right angle, thumb facing the ceiling, shoulder set. Turn your am inwards so that it is close to your stomach.  Exercise: Take the exercise band in your non treatment side and pass it across to your treatment side, ensuring it is on tension.  Exercise: Allow your arm to move outwards towards the midline, controlling the band as you do so. | 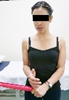 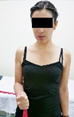 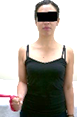 | 1-2 sets of 20 repetitions with 5-10 second rest in between sets. 1-2 times daily.  Progression: Start with arm rotated further inwards so that you have to control the band through greater range.  Progression: Resistance band increased as tolerated |
| **WEEK FOUR** | Posterior muscle strengthening.  Improve strength of posterior shoulder muscles. | **Standing row at 45 degrees**  Start position: Stand up tall, band firmly tied in front of you. Shoulders set. Grasp each end of the band with your hands. Take both arms out to the side to 45 degrees.  Exercise: Pull the upper arms backwards (against the tension of the band (as if to push your elbows backwards). Pull to, but not beyond the body.  **Standing row at 90 degrees**  Start position: Stand up tall, band firmly tied in front of you. Shoulders set. Grasp each end of the band with your hands. Take both arms out to the side to 90 degrees.  Exercise: Pull the upper arms backwards against the tension of the band (as if to push your elbows backwards). Ensure you keep your shoulders up at 90 degrees. | 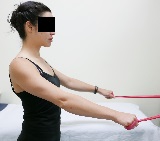 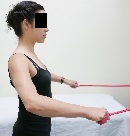    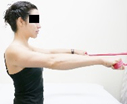 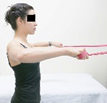 | 1-2 sets of 20 repetitions with 5-10 second rest in between sets. 1-2 times daily.  Progression: Resistance increased as tolerated. |
|  | Flexibility.  Assist in regaining shoulder and thoracic range of motion. | **Anterior shoulder stretch**  Start position: Stand facing corner of room, palms flat against each wall. Ensure low back does not over arch.  Exercise: Gently take chest towards the wall to feel a stretch across the front of the chest (pectoral muscles).  **Thoracic extension in sitting**  Start position: Sitting on chair. Chin gently tucked towards chest. Place finger tips of one hand along breast bone.  Exercise: Gently lift breast bone as if to push fingers up and away, gently extending the mid back. Ensure the low back does not over arch at the same time. | 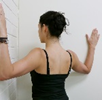  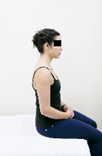 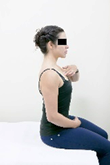 | Hold stretch for 15 seconds and repeat 2-4 times with a 5-10 second rest in between each stretch. I-2 times daily.  1 set of 10 repetitions with 5 second hold and 5-10 second rest in between sets. 1-2 times daily. |
