# Supplementary material for: A comparison of isometric, isotonic concentric and isotonic eccentric exercises in the physiotherapy management of subacromial pain syndrome/rotator cuff tendinopathy: study protocol for a pilot randomised controlled trial
Source: Pilot Feasibility Stud. 2017 Nov 14;3:45. doi: 10.1186/s40814-017-0190-3 (PMC5684744; doi:10.1186/s40814-017-0190-3)
Supplement: Supplementary file 3 — Phase 2 (week 6-12) exercise sheet. (DOCX 457 kb) [file 40814_2017_190_MOESM3_ESM.docx]

*Phase 2 Week 6-12 exercise sheet*

| Scapular retraining. | Motor control:  1-2 sets of 20 repetitions  1 - 2 times per day  5-10 second rest between sets.  Shrug exercise: Resistance increased as tolerated. | 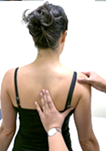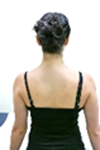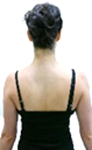 | 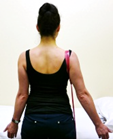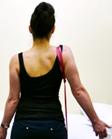 | 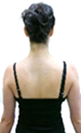 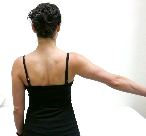 |  |
| --- | --- | --- | --- | --- | --- |
|  |  | **Scapular setting**  Start position: Stand up tall, arm by your side, elbow straight but relaxed.  Exercise: Move shoulder blade into the correct position the physiotherapist has shown you. Hold x 5 seconds. | **Scapular shrug**  Start position: Stand up tall, arm down by your side, elbow straight, with resistance band in position (if you have progressed to this). Move your shoulder blade into the correct position as in the previous exercise.  Exercise: Take your arm out to the side just a little way (20-30 degrees) with your arm rotated so your palm is facing forward. Shrug your shoulder up to your ear, as far as you can, against the resistance of the band if using, but without provoking pain. | **Scapular control in elevation**  Start position: Stand up tall, arm down by your side, elbow straight but relaxed. Move your shoulder blade into the correct position as in the previous exercise.  Exercise: Raise your arm upwards to the side maintaining your corrected shoulder posture. Move only to where you do not provoke pain. With practice you will be able to raise your arm further, while maintaining a good shoulder posture. |  |
| Posterior muscle strengthening.  Ensure you have “set” your shoulder blade before starting each repetition of these exercises.  Safety: Ensure the exercise band is securely fastened to a door knob or other stable object. | 1-2 sets of 20 repetitions  1 - 2 times per day  5-10 second rest between sets.  Resistance increased as tolerated. | **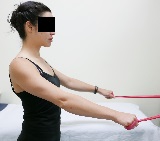 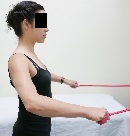** | **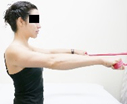 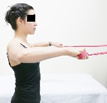** | **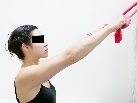 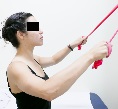** | 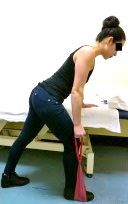 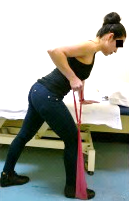 |
|  |  | **Standing row at 45 degrees**  Start position: Stand tall, band firmly tied in front of you. Grasp each end of the band with your hands. Take both arms out to the side to 45 degrees.  Exercise: Pull the upper arms backwards (against the tension of the band (as if to push your elbows backwards). Pull to, but not beyond the body. | **Standing row at 90 degrees**  Start position: Stand tall, band firmly tied in front of you. Grasp each end of the band with your hands. Take both arms out to the side to 90 degrees.  Exercise: Pull the upper arms backwards against the tension of the band (as if to push your elbows backwards). Keep your shoulders up at 90 degrees. | **Standing row at 120 degrees**  Start position: Stand up tall, band firmly tied in front of you. Grasp each end of the band with your hands. Take both arms up so that shoulders are flexed to 120 degrees.  Exercise: Pull upper arms down and backwards against the tension of the band. | **Bent forward row**  Start position: Stand in supported walk standing with the resistance band stabilised under your leading foot, the other end grasped in your hand, band on tension.  Exercise: Pull your arm upwards taking your elbow towards the ceiling, pulling against the tension of the band. |
| Rotator cuff strengthening: shoulder external/internal rotation.  Ensure you have “set” your shoulder blade before starting each repetition of these exercises.  Safety: Ensure the exercise band is securely fastened to a door knob or other stable object. | 1-2 sets of 20 repetitions  1 - 2 times per day  5-10 second rest between sets.  Resistance increased as tolerated. | 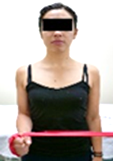 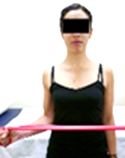 | 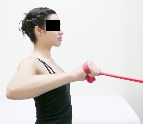 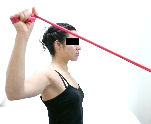 | 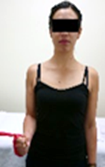 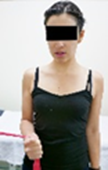 | 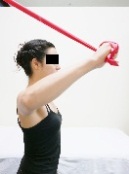 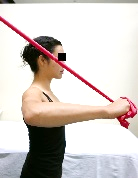 |
|  |  | **External rotation at 0 degrees**  Start position: Stand with your upper arm close to your side, elbow at a right angle and holding the exercise band.  Exercise: Pull the band by turning your forearm outwards. | **External rotation at 90 degrees**  Start position: Stand up tall, band firmly tied in front of you. Grasp the band with your hand (painful shoulder side) and pull it backwards in to the 90 degree standing row end position (see above).  Exercise: With the band on tension, externally rotate your shoulder so that your palm faces frontwards. | **Internal rotation at 0 degrees**  Start position: Stand with your upper arm close to your side, elbow at a right angle and holding the exercise band.  Exercise: Pull the band towards your stomach. | **Internal rotation at 90 degrees**  Start position: Stand with your shoulder and elbow at 90 degrees.  Exercise: Pull against resistance band to take palm towards floor, internally rotating shoulder. |
| Flexibility exercises. | I-2 times daily.  Shoulder stretch: Hold stretch for 15 seconds and repeat 2-4 times with a 5-10 second rest in between each stretch.  Thoracic mobility: 1 set of 10 repetitions with 5 second hold and 5-10 second rest in between sets. | 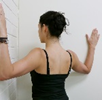 | 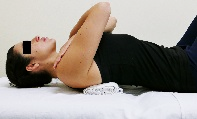 | 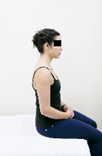 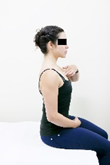 | 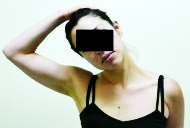 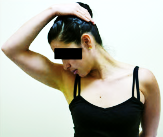 |
|  |  | **Anterior shoulder stretch**  Stand facing corner of room, palms flat against each wall. Ensure low back does not over arch. Gently take chest towards the wall to feel a stretch across the front of the chest (pectoral muscles). | **Thoracic extension in lie**  Lie on back on floor with knees bent and a rolled-up towel or foam roller under upper back. Gently breathe out and let upper back press against the towel/roller and curve upper back gently towards the floor. | **Thoracic extension in sitting**  Sitting on chair. Chin gently tucked towards chest. Place finger tips of one hand along breast bone. Gently lift breast bone as if to push fingers up and away, gently extending the mid back. Ensure the low back does not over arch at the same time. | **Neck stretch (right side shown)**  Sitting in chair with good posture, feet on floor, right arm gently holding the edge of the chair seat.  Tilt head to left shoulder. Place left palm over the side of head and gently pull head a little further towards the left shoulder. Hold. Then turn to look towards the left arm pit. Hold. Ensure you stay relaxed and keep your right shoulder down. Only go to where a gentle stretch is felt in the muscles on the right side of the neck/upper shoulder. |
